# The Status of Musculoskeletal Disorders and Its Influence on the Working Ability of Oil Workers in Xinjiang, China

**DOI:** 10.3390/ijerph15050842

**Published:** 2018-04-24

**Authors:** Hua Ge, Xuemei Sun, Jiwen Liu, Chen Zhang

**Affiliations:** Department of Occupational Health and Environmental Health, College of Public Health, Xinjiang Medical University, Urumqi 830011, China; gehua2710@sina.com (H.G.); sunsun9010@163.com (X.S.)

**Keywords:** musculoskeletal disorders, working ability, oil workers

## Abstract

The purpose of this study was to investigate the status of musculoskeletal disorders (MSDs) and its influence on the working ability of oil workers, and to provide a theoretical basis for helping lessen the burden of MSDs and improve the man-machine environment of oil workers. The cluster sampling method was used to study 2000 workers who had been employed for more than 1 year in this field. We investigated the prevalence rate and the work ability index (WAI). A total of 1935 valid questionnaires were collected, a response rate of 96.75%. There were 1639 people who had suffered from MSDs in the past year, a prevalence rate of 84.7%. The damage detection rate in female oil workers was higher than in males, and the damage detection rate in workers aged 30 to 45 years was higher than that in the other two age groups. The detection rate in less highly-educated oil workers was higher than that in more highly-educated workers. The detection rate in divorced workers was higher than that in other groups. The detection rate in workers between the number of working years of 18 to 25 years was higher than in the other two groups. The detection rate in workers with a high professional title was significantly higher than that in lower-titled workers (*p* < 0.05). The results showed that the WAI scores of the subjects with MSDs were significantly lower than for subjects without MSDs (*p* < 0.05). In a logistic regression analysis, sex, number of working years and WAI index all had an impact on MSDs. We concluded that due to the demands of their role, the oil workers had serious MSDs that influenced their working ability.

## 1. Introduction

Musculoskeletal disorders (MSDs) are common disorders of the muscles, bones, nerves and other systems due to unergonomic working conditions. Low back pain and carpal tunnel syndrome are common. MSDs often occur in the process of workers’ working activities, which not only seriously affect the efficiency of workers, threaten their health and lives, but also bring huge economic burdens to the country and enterprises [1]. Occupational injuries of a musculoskeletal nature have caught the attention of scholars internationally [2,3,4,5] and have been classified as an occupational disease in some developed countries. In occupational diseases confirmed by Eurostat in 2005, WMSDs accounted for 39% of all occupational diseases in EU countries [6]. MSDs are the major health problems and economic burdens in many developed countries, resulting in as many as tens of billions of dollars in compensation for work-related injuries each year [7]. Studies have shown that MSDs in the United States account for 65% of all occupational diseases and have become the fastest growing occupational injury; Most of the costs of occupation-related diseases are caused by the MSDs, which is approximately 130–540 billion dollars/year [8,9]. The annual economic losses caused by MSDs in the Nordic countries are enormous, accounting for 2.7–5.2% of the GNP [10]. MSDs are the most common cause of sick leave or disability in Canada, the United States, Finland, Sweden, and England [11,12].

There are many factors that lead to MSDs. In summary, they can be divided into three categories: The first is professional factors. Studies have shown that long-term repetitive operations, incorrect postures are the main causes of MSDs [13,14]. There are also studies suggesting that long-term repeated lifting, pushing, pulling, and transporting heavy objects increase the incidence of musculoskeletal injuries at the back [15,16]. Long or repeated rotation of the body, bow and other movements, it is likely to make the body in a state of fatigue and difficult to recover, and then cause the back MSD increased significantly [17,18,19]. The second is the individual factor. Factors such as age, gender, and BMI all influence the occurrence of MSDs. Individuals’ lifestyles, such as whether they smoke, exercise, and different levels of education, are also risk factors for MSDs [20]. The third is social and psychological factors. Studies have shown that poor psychosocial factors can induce MSDs [21,22,23]. The National Institute for Occupational Safety and Health (NIOSH) points out that five types of psychosocial factors such as job satisfaction, high workload, work monotony, job control, and social support are related to occupational low back pain and upper extremity musculoskeletal disorders [24].

Studies have found that MSDs will not only impair the health status of the professional population, but also have impact on their ability to work. For example, Ropponen found that musculoskeletal pain impairing work ability [25]. Ge found that the number of working years, neck and shoulder MSD and back MSD of the screen display terminal operator are factors that affect work ability [26]. Another study found that the main factors affecting nurses’ work ability are number of working years, neck and shoulder MSDs, and lower back MSDs. Nurses’ chronic MSDs have effect on the ability to work [27].

The working demands on oil workers are heavy and the work environment is harsh. If workers operate in this environment for a long time, it can be harmful to their physical and mental health. With the modernization of production, the working conditions in petroleum production have been greatly improved [28]. But the nature of oil work is special: Under the effects of working in the field and mostly in isolated desert areas, with drought, high temperatures, strong radiation and other adverse climatic conditions, there is a high prevalence of MSDs in this occupational population. Therefore, the purpose of this survey was to investigate the status of MSDs in oil workers, to provide a theoretical basis for helping lessen the burden of MSDs and improve the man-machine environment of oil workers.

## 2. Material and Methods

### 2.1. Study Subjects

This cross-sectional study was carried out during the period between April and September 2016. Study subjects were employees aged 18–60 years of both sexes oil workers from seven oil companies in Kelamayi City, Xinjiang Autonomous Uygur Region, China. A total of 2000 oil workers who had been working in this field for more than 1 year were recruited using cluster sampling method. After rejecting incomplete and incorrectly completed questionnaires, 1935 subjects were eligible, for an effective valid questionnaire response rate of 96.75%. They were all healthy people without organic diseases, hypertension, mental illness or genetic history of ill-health, and their routine examination (such as blood routine, liver and kidney function, chest X-ray, electrocardiogram, etc.) was normal.

### 2.2. Research Methods

A questionnaire (detailed below) was used to investigate the status of MSDs and its impact on working ability.

#### 2.2.1. General Investigation

This section included general demographic characteristics such as age, sex, ethnicity, marital status, educational level and income, as well as occupational characteristics such as job type, number of working years, title and shift status.

#### 2.2.2. MSDs Investigation

The Nordic Musculoskeletal Disorders Standard Questionnaire [29] was used to collect the data on MSDs status and its effect on working and life in the last year. Physical symptoms such as pain, numbness and inconvenience, and symptoms lasting more than 24 h relating to work, were included, while excluding other medical emergencies and physical disabilities or illness sequelae, etc. The prevalence of MSDs was measured using the prevalence for the last year, i.e., the percentage of respondents who had musculoskeletal symptoms during the previous year.

The method is an indirect method [30], There have been many studies using the method to evaluate workers’ MSDs status [31,32,33]. The advantages of the method are principally its questions is standardized, cost is low, worldwide recognition, and relatively quick identification of the symptoms. However, the complexity on occasions, the statistical treatment of the data and the need to administer questionnaires to a representative portion of the workers under study are the main disadvantages [34].

#### 2.2.3. Working Ability Investigation

The Work Ability Index Questionnaire (WAI) was used to evaluate working ability. This was developed in 1994 by the Finnish National Institute of Occupational Health after years of research and development. It is a test tool used to judge whether a person can finish his/her work continuously. The WAI is used to evaluate changes in the working ability of workers, and the Chinese version has good reliability and validity [35]. The questionnaire is consisted of seven items, including current work ability compared with the lifetime best (0–10), work ability in relation to the demands of the job (score range: 2–10), number of current diseases diagnosed by a physician (score range: 1–7), estimated work impairment due to diseases (score range: 1–6), sick leave during the past 12 months (score range: 1–5), personal prognosis of work ability 2 year from now (score range: 1, 4, or 7), and mental resources (score range: 1–4). Each item is evaluated by different number of questions; therefore, the score ranges of items differ with each other. The total score of WAI is calculated by summing up the scores of all items that ranged from 7–49 points, the greater the score, the stronger the ability to work. Working ability is divided into three levels according to WAI score: 7–27 points (ability to work is poor), 28–36 points (ability to work is medium), and 37 to 49 points (ability to work is high).

### 2.3. Quality Control

In China, workers are required to undergo professional health examinations on a regular basis under the Industrial Safety and Health Act. During the annual professional health examination, we conducted face-to-face interviews with each participant to fill in the questionnaires. To reduce the possibility of bias, before the formal investigation began pilot testing was conducted, checking the reliability of the method. The investigator gave out the questionnaire in person and clarified the purpose, meaning and content of the study. The questionnaire was completed anonymously and collected immediately upon completion.

### 2.4. Statistical Methods

Statistical analysis was performed with SPSS 21.0 (SPSS Inc., Chicago, IL, USA). All measurement data were presented as the mean *±* standard deviation. The *t*-test was used for pairwise comparisons. A Chi-square test was used for the inter-group comparisons. The multivariate analysis was performed by using a logistic regression. The significance level (α) was set at 0.05.

## 3. Results

### 3.1. General Demographic Characteristics of Oil Workers

The average age of the subjects was 37.90 ± 9.19 years, and the average of number of working years was 17.04 ± 11.54 (Table 1).

### 3.2. The Total Prevalence of MSDs symptoms to the Lower Back, Shoulder and Neck

In this survey, 1639 oil workers suffered from MSDs in the last year, the prevalence being 84.7%. Among them, 1593 people suffered from back pain or discomfort (prevalence was 82.33%), 1477 people with neck and shoulder discomfort (prevalence was 76.33%), 5.99% (116) of subjects had hand or arm discomfort, 2.38% (46) had leg or foot discomfort, and 7.63% (148) had discomfort in other parts of the body (Table 2).

### 3.3. Prevalence of MSDs Symptoms of Oil Workers in Different Populations

In the investigation of the prevalence of MSDs in the oil workers, there were significant differences between different sexes, ages, types of work, number of working years, titles, education levels, marital status and incomes (*p* < 0.05). However, no significant difference was observed between different nationalities and shift workers in the prevalence of MSDs (*p* > 0.05). The results showed that the detection rate of disorders in female oil workers was higher than that of males. The detection rate of oil workers’ disorders between the ages of 30–45 years was higher than that of the other two age groups. The detection rate in oil transportation workers was higher than the other two groups. The detection rate in oil workers who had been working for 10 to 25 years was higher than the other two groups. The detection rate in oil workers with high occupational titles was higher than that in lower-status oil workers. The detection rate in oil workers with low qualifications was higher than that in more highly educated workers. The detection rate in divorced oil workers was higher than other groups, and the detection rate in 3000–4500 income oil workers was higher than other groups. (Table 3).

### 3.4. Effects of MSDs on the Working Ability of Oil Workers

The MSDs caused by labor load and posture load of oil workers is bound to lead to a decrease in working ability. The WAI scores of the subjects with MSDs were significantly lower than for the subjects without MSDs (*p* < 0.05). The results showed that MSDs had an influence on workers’ working ability (Table 4).

### 3.5. Exploration of Factors Influencing MSDs

The effects of different population characteristics and working ability on MSDs of oil workers were analyzed by logistic regression. Sex, number of working years, and WAI entered into the regression equation (*p* < 0.05). The results revealed that all these variables had an effect on MSDs (Table 5 and Table 6).

## 4. Discussion

The main clinical manifestations of MSDs are pain and movement disorders. It has a high prevalence among occupational populations. Barzideh reported that 89.9% of nurses have experienced symptoms of these disorders in one or more parts of their musculoskeletal systems in the past 12 years with backache being the most common problem (61.8%) [36]. A study on the crew of MSDs in Venezuelan oil tanker crews showed that the prevalence of MSDs in crews was 82% [1]. The prevalence of MSDs in China was 20~90%, and individual industries even exceed 90% [37]. MSDs not only seriously affects the health status, working ability and quality of life of professional people, but also brings a huge economic burden on the country and society [38].

As the pillar industry of the Chinese national economy, the oil industry has brought economic benefits to the country and the society. Through the exploration, mining, refining, transportation and sale of oil, these companies produce huge economic benefits, increasing the government’s tax revenue and making a great contribution to the development of the country at same time. The petroleum industry is becoming more and more important in the world today. The physical and mental health of the oil workers, and their working ability, are directly related to the development of the oil industry. In the present study, there were differences in the musculoskeletal injuries among the workers with different working abilities, with the detection rate of MSDs being higher in the low ability group. MSDs in the workers have a great influence on their ability to work.

Because of the high workload and hard working conditions, the oil workers are the main population in which MSDs occur. The survey showed that the main areas of MSDs were the back of the waist (83.74%), neck and shoulder (78.01%), or both (75.78%). The results showed that the MSDs suffered by oil workers are serious.

A studies have shown that with age and number of working years, the incidence of MSDs increases, and that there are also differences between men and women [39]. Our results showed that the detection rates of waist, neck and shoulder disorders in female oil workers were higher than that in male workers, which might be related to men’s muscles being more developed than women, with male muscle fiber tissue being thicker and containing less water, so the female musculoskeletal system is more prone to damage than the male one. With respect to type of work, the oil transportation workers’ MSDs detection rate is higher than other types of oil workers. The transportation workers are mainly responsible for conveying crude and refined oil. The working environment is relatively hard, working time is not fixed, and work tasks are heavy. Most of the oil industry implements the shift system. In general, according to the condition of type of work, the distance between the operating area and the urban area, work tasks and so on requires workers to work in the field for a period of time (usually 1 to 6 months) and then go home to rest for some time regularly, then this is repeated. The working conditions for oil workers can be harsh, labor intensity is relatively great, and workers are frequently exposed to harmful working environments such as dust, noise and bad weather conditions. Long-term accumulation of these harmful factors can easily lead to the emergence of some physiological and psychological stress, causing occupational stress [40]. Divorced oil workers’ MSDs were the most serious. The reason might be that after divorce life stresses are relatively great, and the support from the family is relatively low. However, divorced workers not only need to complete the task of work, but also to fulfill their responsibility to the family. In addition, their rest time is relatively lower than other workers [36].

The oil worker’s musculoskeletal system is in a high load condition for a long time and cannot be relieved, causing neck, back pain and other musculoskeletal discomfort. Beyond that, as the oil workers’ work pressure is heavy, work rest time is not regular, and they cannot obtain reasonably early treatment of disorders, over time their working habits and working ability are affected. The external load can cause tissue changes though tissue fatigue, and tissue damage can occur if the load inside the tissue exceeds the mechanical tolerance or the resistance of the tissue structure [41,42,43]. When the muscles and ligaments bear more than the tension threshold, the bone and joint surface can also produce considerable force, resulting in mechanical damage. These injuries can be caused by an excessive pressure, but also may be due to repetitive strain and cannot be alleviated in time. The neck being in flexion, extension, scoliosis and twisting and other bad postures, or repetitive turning and bending, can cause skeletal muscle fatigue, resulting in neck or waist damage, including lesions [44].

Most of the oil exploitation is in the uninhabited desert, where medical and recreational facilities are crude. Oil workers work in harsh conditions and need to face temporary separation from family and friends. Also, many of them are migrant workers, who are not familiar with the surrounding environment and cannot communicate well with their co-workers, and therefore psychological stress is relatively great [45,46]. In addition, due to improper work posture, MSDs often occurs, and the health awareness of oil workers is relatively poor, so few workers will choose to seek medical advice, or cannot get to hospital in a timely manner, compliance is low, not cooperate with the treatment, and all these factors mean that oil workers have a higher prevalence of MSDs. Therefore, to improve the occupational quality of life of the workers, it is necessary to improve their working environment and living security, to enable them to carry out a variety of cultural and recreational activities, and adjust the operating cycle and shift system.

We recognize that there are limitations associated with our study. It is not known if these results can be extrapolated to other regions of China, other countries or other industries. The underlying mechanisms through which MSDs operate on work ability is still not very clear and the study accuracy needed to be verified by perspective studies. In future, further studies with even large sample sizes are needed.

## 5. Conclusions 

In conclusion, this study found due to the demands of their role, the MSDs suffered by oil workers are serious and sex, age, type of work, number of working years, education level, and marital status are the influencing factors. In addition, MSDs had an influence on workers’ working ability. Measures should be taken to help lessen the burden on MSDs and improve the man-machine environment of oil workers.

## Figures and Tables

**Table 1:** The oil workers’ population characteristics.

Items	Groups	Case Number	Percentage (%)
Sex	Male	1078	55.7
	Female	857	44.3
Ethnicity	Han	1527	78.9
	Uygur	302	15.6
	Other	106	5.5
Age (years)	<30	573	29.6
	30–45	914	47.2
	>45	448	23.2
Type of work	Oil production workers	588	30.4
	Oil transportation workers	340	17.6
	Oil refining workers	1007	52.0
Number of working years	<10	763	39.4
	10–25	549	28.4
	>25	623	32.2
Title	None	657	34.0
	Primary	311	16.1
	Intermediate	344	17.8
	Deputy senior and advanced	623	32.2
Work shift	Fixed day shift	717	37.1
	Work in shifts	689	35.6
	Other	529	27.3
Education level	Secondary technical school or college and less	1471	76.0
	University and above	464	24.0
Marital status	Unmarried	428	22.1
	Married	1374	71.0
	Divorced	117	6.0
	Widowed	16	0.8
Income (yuan)	<3000	628	32.5
	3000–4500	880	45.5
	≥4500	427	22.1

**Table 2:** The oil workers’ MSDs symptoms.

Disorders Site	No Damage (%)	Damage (%)
MSDs	296 (15.29)	1639 (84.70)
Waist and back	342 (17.67)	1593 (82.33)
Neck and shoulder	458 (23.67)	1477 (76.33)
Hand/arm	1819 (94.00)	116 (5.99)
Leg/foot	1889 (97.62)	46 (2.38)
Others	1787 (92.35)	148 (7.63)

**Table 3:** Prevalence of MSDs symptoms of Oil Workers in Different Populations.

Items	Groups	Low Back Pain (%)	Chi-Square Value	*p*-Value	Neck Shoulder Pain (%)	Chi-Square Value	*p*-Value
Sex	Male	846 (78.47)	24.75	<0.01	751 (69.66)	33.76	<0.01
	Female	747 (87.16)			696 (81.21)		
Ethnicity	Han	1264 (82.77)	1.57	0.456	1147 (75.11)	1.06	0.588
	Uygur	241 (79.80)			219 (72.51)		
	Other	88 (83.01)			81 (76.41)		
Age (years)	<30	435 (75.92)	25.22	<0.01	371 (64.75)	51.19	<0.01
	30–45	787 (86.11)			743 (81.29)		
	>45	371 (82.81)			333 (74.33)		
Type of work	Oil production workers	325 (55.27)	42.58	<0.01	517 (87.92)	83.6	<0.01
	Oil transportation workers	280 (82.64)			252 (74.12)		
	Oil refining workers	781 (77.56)			678 (67.32)		
Number of working years	<10	599 (78.50)	12.76	0.002	521 (68.28)	32.98	<0.01
	10–25	468 (85.24)			450 (81.96)		
	>25	526 (84.43)			476 (76.40)		
Title	None	507 (77.17)	21.18	<0.01	441 (67.12)	32.32	<0.01
	Primary	266 (85.53)			243 (78.13)		
	Intermediate	282 (81.97)			264 (76.74)		
	Deputy senior and advanced	538 (86.35)			499 (80.09)		
Work shift	Fixed day shift	587 (81.86)	0.163	0.992	526 (73.36)	1.234	0.54
	Work in shifts	569 (82.58)			520 (75.47)		
	Other	437 (82.61)			401 (75.80)		
Education level	Secondary technical school or college and less	1232 (83.75)	8.59	0.003	1124 (76.41)	8.69	0.003
	University and above	361 (77.80)			323 (69.61)		
Marital status	Unmarried	315 (73.59)	37.49	<0.01	274 (64.01)	46.92	<0.01
	Married	1158 (84.28)			1059 (77.07)		
	Divorced	109 (93.16)			105 (89.74)		
	Widowed	11 (68.75)			9 (56.25)		
Income (yuan)	<3000	477 (75.95)	28.62	<0.01	409 (65.12)	50.15	<0.01
	3000–4500	762 (86.59)			714 (81.13)		
	≥4500	354 (82.90)			324 (75.87)		

**Table 4:** Comparison of WAI scores in those with and without musculoskeletal injuries.

Index	Low Back Pain		Neck and Shoulder Pain	
	Not Sick	Sick	Not Sick	Sick
WAI score	36.46 ± 4.76	32.58 ± 5.21	39.53 ± 5.19	32.37 ± 5.09
*t* value	11.01		11.639	
*p*-value	<0.01		<0.01	

Notes: WAI = Work Ability Index.

**Table 5:** Assignment of factors specific points.

Variable	Name	Assignment
y	MSDs	0 = no, 1 = yes
x1	Sex	1 = male, 2 = female
x2	Age	1 = <30 years, 2 = 30–45 years, 3 = >45 years
x3	Education level	1 = college and above, 2 = college or lower
x4	Marital status	1 = unmarried, 2 = married, 3 = divorced, 4 = widowed, 5 = remarried
x5	Income	1 = <3000 yuan 2 = 3000–4000 yuan, 3 = >4500 yuan
x6	Type of work	1 = drilling, 2 = oil, 3 = oil recovery
x7	Number of working years	1 = <10 years, 2 = 10–25 years, 3 = >25 years
x8	Title	1 = none, 2 = primary, 3 = intermediate, 4 = vice advanced and advanced
x9	The Work Ability Index (WAI)	1 = poor working ability, 2 = work ability, 3 = high working ability

**Table 6:** The effects of oil workers’ MSDs factors according to the results of the logistic regression analysis.

Variable	β	OR	95%CI	Chi-Square Value	*p*-Value
Sex	0.586	1.796	1.365–2.364	17.486	<0.01
Age	0.012	1.012	0.978–1.046	0.457	0.499
Education level	−0.22	0.803	0.587–1.098	1.885	0.17
Marital status	0.221	0.802	0.326–1.970	0.231	0.63
Income	0.03	1.031	0.833–1.276	0.077	0.782
Type of work	−0.199	0.82	0.578–1.163	1.237	0.266
Number of working years	−0.421	0.656	0.555–0.775	24.36	<0.01
Title	0.047	1.049	0.929–1.184	0.588	0.443
WAI	−0.974	0.377	0.293–0.486	6.955	<0.01

Notes: OR = Odds ratios; 95% CI = 95% confidence intervals.
